# Crystal structure of barium dinickel(II) iron(III) tris­[orthophosphate(V)], BaNi_2_Fe(PO_4_)_3_

**DOI:** 10.1107/S2056989023000336

**Published:** 2023-01-17

**Authors:** Said Ouaatta, Adam Bouraima, Elhassan Benhsina, Jamal Khmiyas, Abderrazzak Assani, Mohamed Saadi, Lahcen El Ammari

**Affiliations:** a Laboratoire de Chimie Appliquée des Matériaux, Centre des Sciences des Matériaux, Faculty of Science, Mohammed V University in Rabat, Avenue Ibn Batouta, BP 1014, Rabat, Morocco; b Laboratoire de Chimie des Matériaux Inorganiques, Faculté des Sciences, Département de Chimie, Université des Sciences et Techniques de Masuku, BP 943, Franceville, Gabon; Vienna University of Technology, Austria

**Keywords:** crystal structure, orthophosphates, solid-state reactions, α-CrPO_4_-type structure BaNi_2_Fe(PO_4_)_3_

## Abstract

The orthophosphate BaNi_2_Fe(PO_4_)_3_ has been synthesized by a solid-state reaction route and characterized by single-crystal X-ray diffraction and energy-dispersive X-ray spectroscopy. The crystal structure comprises (100) sheets made up of [Ni_2_O_10_] dimers that are linked to two PO_4_ tetra­hedra *via* common edges and vertices and of linear infinite [010] chains of corner-sharing [FeO_6_] octa­hedra and [PO_4_] tetra­hedra. The linkage of the sheets and chains into a framework is accomplished through common vertices of PO_4_ tetra­hedra and [FeO_6_] octa­hedra. The framework is perforated by channels in which positionally disordered Ba^2+^ cations are located.

## Abstract 2

The orthophosphate BaNi_2_Fe(PO_4_)_3_ crystallizes in the α-CrPO_4_ type of structure, in which edge-sharing [Ni_2_O_10_] octa­hedra are linked to PO_4_ tetra­hedra and [FeO_6_] octa­hedra to form a three-dimensional framework delimiting channels which house disordered Ba^2+^ cations.

## 1. Chemical context

Phosphate-based materials have been studied extensively in the past. Among them are orthophosphates, which have gained great inter­est in recent years owing to their structural richness (Maeda, 2004 ▸) and their promising applications, for example in electrochemical catalysis (Dwibedi *et al.*, 2020 ▸; Cheng *et al.*, 2021 ▸; Rekha *et al.*, 2021 ▸; Anahmadi *et al.*, 2022 ▸). Furthermore, orthophosphates doped with rare-earth cations have shown excellent optical properties (Ci *et al.*, 2014 ▸; Li *et al.*, 2021 ▸; Indumathi *et al.*, 2022 ▸), along with a wide range of applications for use in luminescence emission displays (Li *et al.*, 2008 ▸; Wan *et al.*, 2010 ▸; Yang *et al.*, 2019 ▸; Santos *et al.*, 2022 ▸).

In this context, our research inter­est is connected with tris-orthophosphate-based materials with general formula (*A*_2_/*B*)*M*_2_ *M*′(PO_4_)_3_, where *A* can be an alkali, *B* an alkaline earth and *M* and *M*′ transition metal cations. The crystal structures of these orthophosphates adopt the α-CrPO_4_ type of structure, consisting of a three-dimensional framework made up of [*M*O_6_] and [*M*′O_6_] octa­hedra sharing corners and/or edges with PO_4_ tetra­hedra. This framework is permeated by channels in which the *A* or *B* cations are located.

We report herein on the synthesis and structural characterization of barium dinickel(II) iron(III) tris-orthophosphate, BaNi_2_Fe(PO_4_)_3_.

## 2. Structural commentary

**Figure 1:**
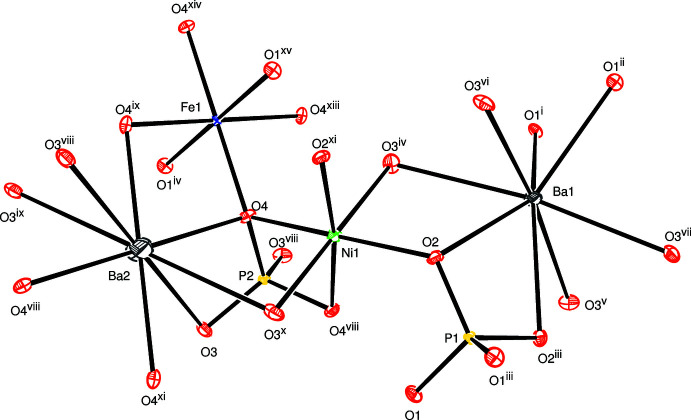
The principal building units in the crystal structure of the title compound. Displacement ellipsoids are drawn at the 50% probability level. [Symmetry codes: (i) −*x* + 1, −*y* + 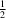 , *z* − 1; (ii) *x*, *y*, *z* − 1; (iii) −*x* + 1, −*y* + 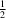 , *z*; (iv) −*x* + 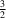 , −*y* + 1, *z* − 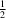 ; (v) *x* − 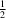 , *y* − 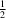 , *z* − 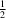 ; (vi) −*x* + 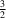 , *y* − 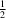 , *z* − 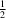 ; (vii) *x* − 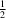 , −*y* + 1, *z* − 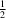 ; (viii) −*x* + 2, −*y* + 1, −*z* + 2; (ix) −*x* + 2, *y*, *z*; (x) *x*, −*y* + 1, −*z* + 2; (xi) −*x* + 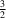 , −*y* + 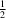 , −*z* + 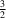 ; (xii) −*x* + 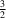 , *y*, −*z* + 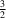 ; (xiii) *x*, −*y* + 1, −*z* + 1; (xiv) −*x* + 2, −*y* + 1, −*z* + 1; (xv) *x* + 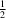 , *y*, −*z* + 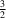 ].

**Figure 2:**
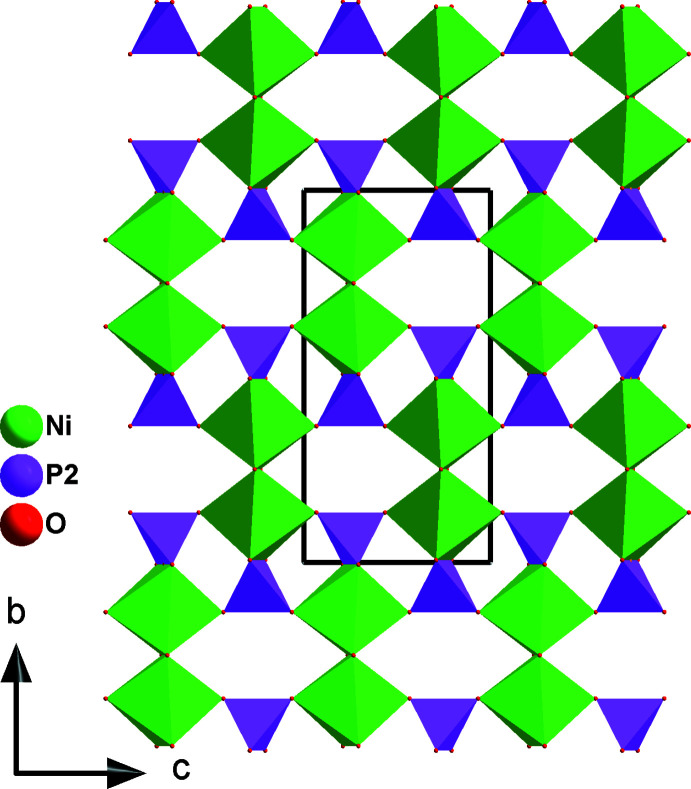
Projection of a (100) sheet along [100] showing the [Ni_2_P(2)_2_O_14_] unit.

**Figure 3:**
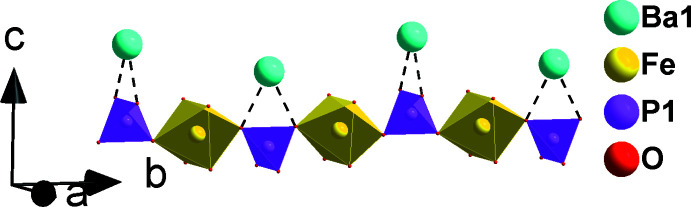
A chain formed by sharing corners of [FeO_6_] octa­hedra and P1O_4_ tetra­hedra, alternating with a zigzag arrangement of barium cations (Ba1) along [010].

**Figure 4:**
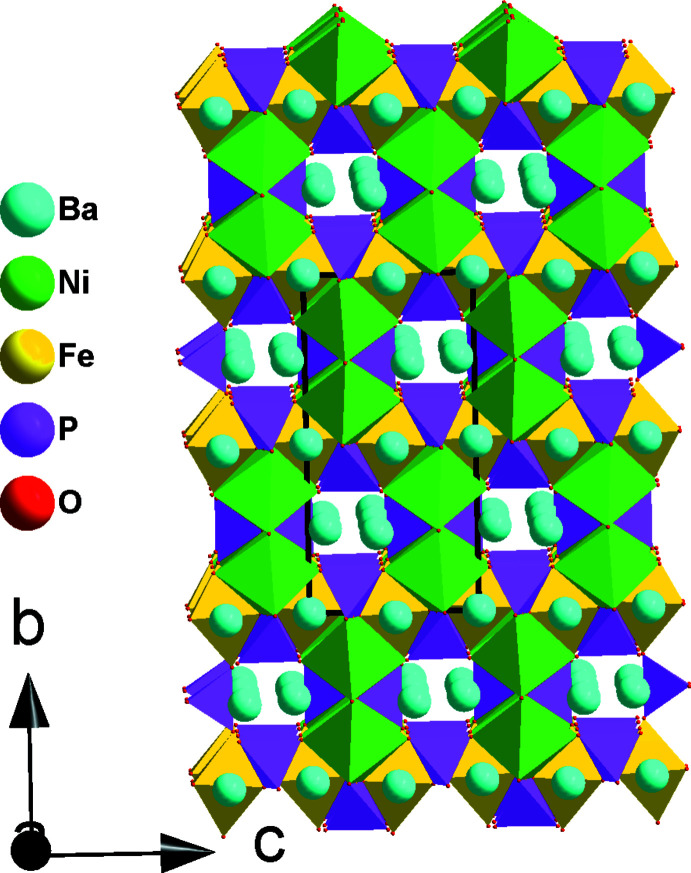
Polyhedral representation of the crystal structure of BaNi_2_Fe(PO_4_)_3_ showing Ba1 in the channels running along the [100] direction and a row of underoccupied Ba2 along [001].

The title compound is related to the strontium and calcium homologs *M*Ni_2_Fe(PO_4_)_3_ (*M* = Sr, Ca; Ouaatta *et al.*, 2015 ▸, 2017 ▸), all adopting the α-CrPO_4_ structure type (Attfield *et al.*, 1986 ▸). The asymmetric unit of BaNi_2_Fe(PO_4_)_3_ is comprised of ten sites, eight of which are on special positions, except the O3 and O4 sites on a general position (Wyckoff position 16 *j*). Ba1 (site occupation 0.9868) exhibits site symmetry *mm*2 (4 *e*), Ba2 (site occupation 0.0132) 2/*m* (4 *a*), Fe1 2/*m* (4 *b*), Ni1 2 (8 *g*), P1 *mm*2 (4 *e*), P2 2 (8 *g*), while O1 and O2 occupy sites with *m* (8 *h*) and *m* (8 *i*) symmetry, respectively. The framework structure of BaNi_2_Fe(PO_4_)_3_ is composed of extended (100) sheets and linear infinite chains extending parallel to [010] (Fig. 1 ▸). The (100) sheets are made up from edge-sharing [Ni_2_O_10_] dimers linked to two P2O_4_ tetra­hedra *via* common edges to form an [Ni_2_P2_2_O_14_] unit that is linked to four neighboring units (Fig. 2 ▸). Between these sheets appear the linear infinite chains resulting from the alternating linkage of P1O_4_ tetra­hedra and [FeO_6_] octa­hedra, which are surrounded by a zigzag arrangement of Ba^2+^ cations (Fig. 3 ▸). The sheets and chains are linked through common vertices of PO_4_ tetra­hedra and [FeO_6_] octa­hedra into a framework, which delimits two types of channels parallel to [100] and [010] in which the disordered Ba^2+^ cations are located (Figs. 4 ▸, 5 ▸).

To confirm the structure model of BaNi_2_Fe(PO_4_)_3_, the bond-valence method (Brown, 1977 ▸; 1978 ▸; Brown & Altermatt, 1985 ▸) and charge distribution (CHARDI) concept (Hoppe *et al.*, 1989 ▸) were employed by making use of the programs *EXPO2014* (Altomare *et al.*, 2013 ▸) and *CHARDI2015* (Nespolo & Guillot, 2016 ▸), respectively. Table 1 ▸ compiles all cationic valences *V*(*i*) computed with the bond-valence method and their related charges *Q*(*i*) obtained with the CHARDI concept. The resulting *Q*(*i*) and *V*(*i*) values are all close to the corresponding charges *q*(*i*)×sof(*i*) [*q*(*i*) are formal oxidation numbers weighted by the site occupation factors sof(*i*)]. In summary, the expected oxidation states of Ba^2+^, Ni^2+^, Fe^3+^ and P^5+^ are predicted through the charge distribution. The inter­nal criterion *q*(*i*)/*Q*(*i*) is very near to 1 for all ionic species and the mean absolute percentage deviation (MAPD), which gives a measure for the agreement between the *q*(*i*) and *Q*(*i*) charges, is just 1.3%, thus confirming the validity of the structure model (Eon & Nespolo, 2015 ▸). The global instability index (GII; Salinas-Sanchez *et al.*, 1992 ▸) of 0.13 is a further confirmation of the structure model.

## 3. Database survey

It is reasonable to compare the crystal structure of the title compound with that of α-CrPO_4_ (Glaum *et al.*, 1986 ▸). Both phosphates crystallize in the ortho­rhom­bic system in space group type *Imma*. Their unit-cell parameters are nearly the same despite the differences between their chemical formulae. In the structure of α-CrPO_4_, the Cr^3+^ and P^5+^ cations occupy four special positions that are part of a framework is comprised of [CrO_6_] octa­hedra and [PO_4_] tetra­hedra. The resultant framework is permeated by vacant channels along [100] and [010]. The formula of α-CrPO_4_ can be written as *X*1*X*2Cr1Cr2_2_(PO_4_)_3_, where *X*1, *X*2 represent the empty channel sites. Accordingly, the substitution of Cr1 or Cr2 by a divalent cation requires charge compensation by cations located in the channels to result in *AA*’*MM*’_2_(PO_4_)_3_ compounds such as BaNi_2_Fe(PO_4_)_3_, or *M*Ni_2_Fe(PO_4_)_3_ (*M* = Sr, Ca; Ouaatta *et al.*, 2015 ▸, 2017 ▸). The difference between BaNi_2_Fe(PO_4_)_3_ and the closely related *M*Ni_2_Fe(PO_4_)_3_ structures pertains to the *M* site, which is split into two sites for the title compound and fully occupied for *M* = Ca, Sr.

## 4. Synthesis and crystallization

BaNi_2_Fe(PO_4_)_3_ was prepared from a mixture of Ba(NO_3_)_2_ (Merck, 98.5%), Ni(NO_3_)_2_·6H_2_O (Riedel-de-Haén, 97%), Fe(NO_3_)_3_·9H_2_O (Panreac, 98%) and H_3_PO_4_ (85%_wt_) in the molar ratio of Ba:Ni:Fe:P = 1:2:1:3. The precursors were suspended in 50 ml of distilled water and stirred without warming for 24 h before heating to dryness at 373 K. The obtained dry residue was ground in an agate mortar until homogeneous, subsequently heated in a platinum crucible up to 673 K to remove volatile decomposition products, and then melted at 1433 K. After being kept at this temperature for one h, the melt was cooled down slowly at a rate of 5 K h^−1^ to 1233 K and then to room temperature. Single crystals with a brown color and different forms were obtained after leaching with distilled water.

**Figure 6:**
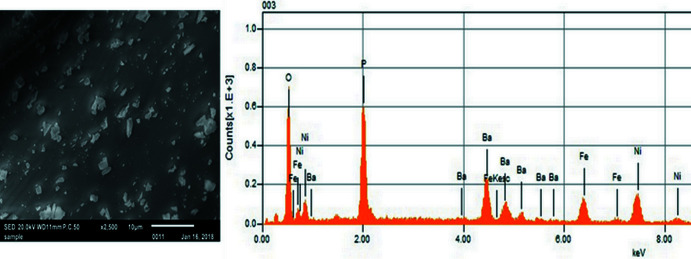
SEM micrograph and results of an EDS measurement of the title compound.

Chemical analysis of the title phosphate was performed with an energy-dispersive X-ray spectroscopy (EDS) microprobe mounted on a JEOL JSM-IT100 in TouchScope^TM^ scanning electron microscope. The EDS spectrum is depicted in Fig. 6 ▸ and confirms the presence of only barium, nickel, iron, phospho­rus and oxygen in approximately the correct ratios, as shown in Table 2 ▸.

## 5. Refinement

Crystal data, data collection and structure refinement details are summarized in Table 3 ▸. After assignment of the atomic sites according to the related *M*Ni_2_Fe(PO_4_)_3_ structures (*M* = Sr, Ca; Ouaatta *et al.*, 2015 ▸, 2017 ▸), a difference-Fourier map revealed a maximum electron density of 3.61 Å^−3^ that was finally modeled as a considerably underoccupied Ba site (Ba2). For the final model, the sum of site-occupation factors for the Ba1 and Ba2 sites were constrained to be 1. The highest remaining maximum and minimum electronic densities are 0.59 Å and 0.47 Å from Ba1 and Ni1, respectively.

## Supplementary Material

Crystal structure: contains datablock(s) I. DOI: 10.1107/S2056989023000336/wm5667sup1.cif

Structure factors: contains datablock(s) I. DOI: 10.1107/S2056989023000336/wm5667Isup2.hkl

CCDC reference: 2172184

Additional supporting information:  crystallographic information; 3D view; checkCIF report

## Figures and Tables

**Figure 5:**
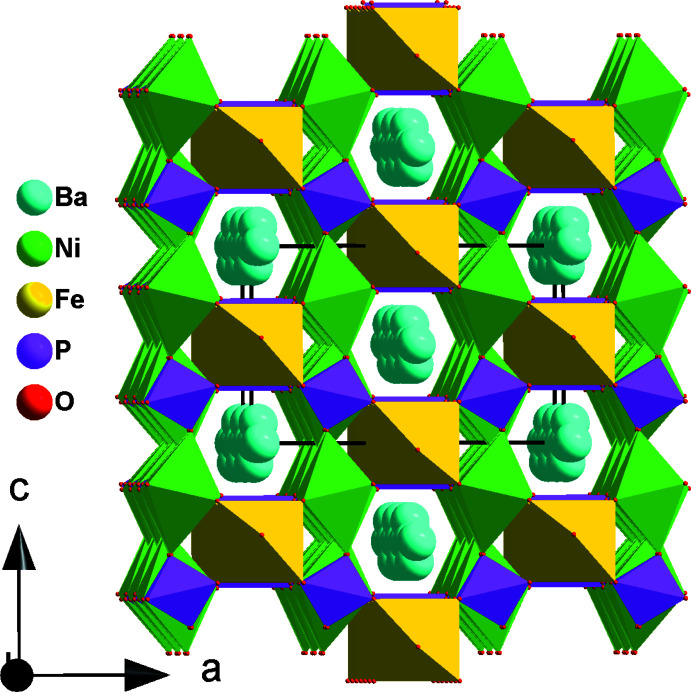
Polyhedral representation of the crystal structure of BaNi_2_Fe(PO_4_)_3_ showing Ba1 and Ba2 in the channels.

**Table 1:** Bond valence and CHARDI analyses for the cations in the title compound *q*(*i*) = formal oxidation number; sof(*i*) = site occupancy; CN(*i*) = classical coordination number; *Q*(*i*) = calculated charge; *V*(*i*) = calculated valence; ECoN(*i*) = effective coordination number.

Cation	*q*(*i*)	sof(*i*)	CN(*i*)	ECoN(*i*)	*V*(*i*)	*Q*(*i*)	*q*(*i*)/*Q*(*i*)
Ba1	1.98	0.99	8	7.99	2.37	1.98	1.00
Ba2	0.02	0.01	8	5.43	0.02	0.99	
Ni	2.00	1.00	6	5.97	2.00	1.98	1.01
Fe	3.00	1.00	4	5.96	3.01	2.99	1.00
P1	5.00	1.00	4	3.99	4.95	4.83	1.04
P2	5.00	1.00	4	3.96	4.85	5.11	0.98

**Table 2:** Atom percentages in BaNi_2_Fe(PO_4_)_3_ as determined by EDS

Element	Atomic percentage	Sigma
O	56.74	0.13
P	19.60	0.16
Fe	5.63	0.17
Ni	12.25	0.27
Ba	5.78	0.30
Total	100.00	

**Table 3:** Experimental details

Crystal data	
Chemical formula	BaNi_2_Fe(PO_4_)_3_
*M*_r_	595.52
Crystal system, space group	Orthorhombic, *I* *m* *m* *a*
Temperature (K)	296
*a*, *b*, *c* (Å)	10.4711 (2), 13.2007 (3), 6.6132 (1)
*V* (Å^3^)	914.12 (3)
*Z*	4
Radiation type	Mo *K*α
μ (mm^−1^)	10.46
Crystal size (mm)	0.32 × 0.25 × 0.19

Data collection	
Diffractometer	Bruker X8 *APEX* Diffractometer
Absorption correction	Multi-scan (*SADABS*; Krause *et al.*, 2015 ▸)
*T*_min_, *T*_max_	0.624, 0.748
No. of measured, independent and observed [*I* > 2σ(*I*)] reflections	18099, 1460, 1440
*R*_int_	0.029
(sin θ/λ)_max_ (Å^−1^)	0.893

Refinement	
*R*[*F* ^2^ > 2σ(*F* ^2^)], *wR*(*F* ^2^), *S*	0.015, 0.036, 1.21
No. of reflections	1460
No. of parameters	58
Δρ_max_, Δρ_min_ (e Å^−3^)	1.33, −0.78

Computer programs: *APEX3* (Bruker, 2016 ▸), *SAINT* (Bruker, 2016 ▸), *SAINT* (Bruker, 2016 ▸), *SHELXT2014/4* (Sheldrick, 2015*a* ▸), *SHELXL2018/3* (Sheldrick, 2015*b* ▸), *ORTEP-3 for Windows* (Farrugia, 2012 ▸), *DIAMOND* (Brandenburg, 2006 ▸), *publCIF* (Westrip, 2010 ▸).

## supplementary crystallographic information

### Crystal data

**Table:**

BaNi_2_Fe(PO_4_)_3_	*D*_x_ = 4.327 Mg m^−^^3^
*M**_r_* = 595.52	Mo *K*α radiation, λ = 0.71073 Å
Orthorhombic, *I**m**m**a*	Cell parameters from 1460 reflections
*a* = 10.4711 (2) Å	θ = 3.1–39.4°
*b* = 13.2007 (3) Å	µ = 10.46 mm^−^^1^
*c* = 6.6132 (1) Å	*T* = 296 K
*V* = 914.12 (3) Å^3^	Block, colourless
*Z* = 4	0.32 × 0.25 × 0.19 mm
*F*(000) = 1116	

### Data collection

**Table:**

Bruker X8 APEX Diffractometer	1460 independent reflections
Radiation source: fine-focus sealed tube	1440 reflections with *I* > 2σ(*I*)
Graphite monochromator	*R*_int_ = 0.029
φ and ω scans	θ_max_ = 39.4°, θ_min_ = 3.1°
Absorption correction: multi-scan (SADABS; Krause *et al.*, 2015)	*h* = −18→13
*T*_min_ = 0.624, *T*_max_ = 0.748	*k* = −23→23
18099 measured reflections	*l* = −11→11

### Refinement

**Table:**

Refinement on *F*^2^	Primary atom site location: structure-invariant direct methods
Least-squares matrix: full	Secondary atom site location: difference Fourier map
*R*[*F*^2^ > 2σ(*F*^2^)] = 0.015	*w* = 1/[σ^2^(*F*_o_^2^) + (0.0147*P*)^2^ + 1.8221*P*] where *P* = (*F*_o_^2^ + 2*F*_c_^2^)/3
*wR*(*F*^2^) = 0.036	(Δ/σ)_max_ = 0.001
*S* = 1.21	Δρ_max_ = 1.33 e Å^−^^3^
1460 reflections	Δρ_min_ = −0.78 e Å^−^^3^
58 parameters	Extinction correction: *SHELXL2018/3* (Sheldrick, 2015b), Fc^*^=kFc[1+0.001xFc^2^λ^3^/sin(2θ)]^-1/4^
0 restraints	Extinction coefficient: 0.00403 (16)

### Special details

**Table:**

Geometry. All esds (except the esd in the dihedral angle between two l.s. planes) are estimated using the full covariance matrix. The cell esds are taken into account individually in the estimation of esds in distances, angles and torsion angles; correlations between esds in cell parameters are only used when they are defined by crystal symmetry. An approximate (isotropic) treatment of cell esds is used for estimating esds involving l.s. planes.

### Fractional atomic coordinates and isotropic or equivalent isotropic displacement parameters (Å^2^)

**Table:**

	*x*	*y*	*z*	*U*_iso_*/*U*_eq_	Occ. (<1)
Ba1	0.500000	0.250000	0.39902 (2)	0.00770 (4)	0.9868
Ba2	1.000000	0.500000	1.000000	0.020 (2)	0.0132
Ni1	0.750000	0.36701 (2)	0.750000	0.00501 (4)	
Fe1	1.000000	0.500000	0.500000	0.00337 (5)	
P1	0.500000	0.250000	0.90454 (8)	0.00317 (8)	
P2	0.750000	0.57020 (3)	0.750000	0.00365 (6)	
O1	0.500000	0.34524 (8)	1.03493 (17)	0.00581 (16)	
O2	0.61939 (10)	0.250000	0.76536 (17)	0.00534 (15)	
O3	0.78276 (8)	0.63385 (6)	0.93423 (13)	0.00771 (12)	
O4	0.86254 (7)	0.49395 (6)	0.70839 (12)	0.00574 (11)	

### Atomic displacement parameters (Å^2^)

**Table:**

	*U*^11^	*U*^22^	*U*^33^	*U*^12^	*U*^13^	*U*^23^
Ba1	0.00681 (5)	0.01253 (6)	0.00377 (5)	0.000	0.000	0.000
Ba2	0.014 (5)	0.041 (8)	0.006 (4)	0.000	0.000	0.000 (4)
Ni1	0.00498 (7)	0.00383 (7)	0.00621 (8)	0.000	0.00057 (5)	0.000
Fe1	0.00303 (10)	0.00352 (10)	0.00355 (10)	0.000	0.000	0.00008 (8)
P1	0.00348 (18)	0.00281 (17)	0.00322 (18)	0.000	0.000	0.000
P2	0.00380 (13)	0.00392 (13)	0.00324 (12)	0.000	0.00055 (10)	0.000
O1	0.0078 (4)	0.0033 (3)	0.0063 (4)	0.000	0.000	−0.0014 (3)
O2	0.0042 (4)	0.0063 (4)	0.0055 (4)	0.000	0.0016 (3)	0.000
O3	0.0096 (3)	0.0080 (3)	0.0055 (3)	−0.0024 (2)	0.0007 (2)	−0.0023 (2)
O4	0.0045 (3)	0.0058 (3)	0.0070 (3)	0.0009 (2)	0.0016 (2)	0.0006 (2)

### Geometric parameters (Å, º)

**Table:**

Ba1—O1^i^	2.7163 (11)	Ni1—O4	2.0670 (8)
Ba1—O1^ii^	2.7163 (11)	Ni1—O4^xii^	2.0670 (8)
Ba1—O2^iii^	2.7262 (11)	Ni1—O3^x^	2.1163 (8)
Ba1—O2	2.7262 (11)	Ni1—O3^iv^	2.1163 (8)
Ba1—O3^iv^	2.7530 (8)	Fe1—O4	1.9944 (8)
Ba1—O3^v^	2.7530 (8)	Fe1—O4^ix^	1.9944 (8)
Ba1—O3^vi^	2.7530 (8)	Fe1—O4^xiii^	1.9944 (8)
Ba1—O3^vii^	2.7530 (8)	Fe1—O4^xiv^	1.9944 (8)
Ba2—O4	2.4077 (8)	Fe1—O1^iv^	2.0559 (11)
Ba2—O4^viii^	2.4078 (8)	Fe1—O1^xv^	2.0559 (11)
Ba2—O4^ix^	2.4078 (8)	P1—O1	1.5246 (11)
Ba2—O4^x^	2.4078 (8)	P1—O1^iii^	1.5246 (11)
Ba2—O3^ix^	2.9129 (9)	P1—O2	1.5524 (11)
Ba2—O3^x^	2.9129 (9)	P1—O2^iii^	1.5524 (11)
Ba2—O3^viii^	2.9129 (9)	P2—O3	1.5192 (8)
Ba2—O3	2.9129 (9)	P2—O3^xii^	1.5193 (8)
Ni1—O2^xi^	2.0655 (7)	P2—O4^xii^	1.5739 (8)
Ni1—O2	2.0655 (7)	P2—O4	1.5739 (8)
			
O1^i^—Ba1—O1^ii^	55.14 (4)	O4^viii^—Ba2—O3	124.49 (2)
O1^i^—Ba1—O2^iii^	141.97 (2)	O4^ix^—Ba2—O3	111.55 (2)
O1^ii^—Ba1—O2^iii^	141.97 (2)	O4^x^—Ba2—O3	68.45 (2)
O1^i^—Ba1—O2	141.97 (2)	O3^ix^—Ba2—O3	102.68 (3)
O1^ii^—Ba1—O2	141.97 (2)	O3^x^—Ba2—O3	77.32 (3)
O2^iii^—Ba1—O2	54.59 (5)	O3^viii^—Ba2—O3	180.0
O1^i^—Ba1—O3^iv^	109.44 (2)	O2^xi^—Ni1—O2	83.20 (5)
O1^ii^—Ba1—O3^iv^	79.47 (2)	O2^xi^—Ni1—O4	102.84 (3)
O2^iii^—Ba1—O3^iv^	107.68 (3)	O2—Ni1—O4	172.00 (3)
O2—Ba1—O3^iv^	63.00 (2)	O2^xi^—Ni1—O4^xii^	172.00 (4)
O1^i^—Ba1—O3^v^	79.47 (2)	O2—Ni1—O4^xii^	102.84 (3)
O1^ii^—Ba1—O3^v^	109.44 (2)	O4—Ni1—O4^xii^	71.66 (4)
O2^iii^—Ba1—O3^v^	63.00 (2)	O2^xi^—Ni1—O3^x^	86.40 (4)
O2—Ba1—O3^v^	107.68 (3)	O2—Ni1—O3^x^	93.14 (4)
O3^iv^—Ba1—O3^v^	170.30 (4)	O4—Ni1—O3^x^	92.48 (3)
O1^i^—Ba1—O3^vi^	79.47 (2)	O4^xii^—Ni1—O3^x^	88.02 (3)
O1^ii^—Ba1—O3^vi^	109.44 (2)	O2^xi^—Ni1—O3^iv^	93.14 (4)
O2^iii^—Ba1—O3^vi^	107.68 (3)	O2—Ni1—O3^iv^	86.40 (4)
O2—Ba1—O3^vi^	63.00 (2)	O4—Ni1—O3^iv^	88.02 (3)
O3^iv^—Ba1—O3^vi^	67.69 (4)	O4^xii^—Ni1—O3^iv^	92.48 (3)
O3^v^—Ba1—O3^vi^	111.43 (4)	O3^x^—Ni1—O3^iv^	179.39 (5)
O1^i^—Ba1—O3^vii^	109.44 (2)	O2^xi^—Ni1—P2	138.40 (2)
O1^ii^—Ba1—O3^vii^	79.47 (2)	O2—Ni1—P2	138.40 (2)
O2^iii^—Ba1—O3^vii^	63.00 (2)	O4—Fe1—O4^ix^	92.40 (5)
O2—Ba1—O3^vii^	107.68 (3)	O4—Fe1—O4^xiii^	87.60 (5)
O3^iv^—Ba1—O3^vii^	111.43 (4)	O4^ix^—Fe1—O4^xiii^	180.0
O3^v^—Ba1—O3^vii^	67.69 (4)	O4—Fe1—O4^xiv^	180.0
O3^vi^—Ba1—O3^vii^	170.30 (4)	O4^ix^—Fe1—O4^xiv^	87.60 (5)
O4—Ba2—O4^viii^	180.0	O4^xiii^—Fe1—O4^xiv^	92.40 (5)
O4—Ba2—O4^ix^	73.43 (4)	O4—Fe1—O1^iv^	87.83 (3)
O4^viii^—Ba2—O4^ix^	106.57 (4)	O4^ix^—Fe1—O1^iv^	87.83 (3)
O4—Ba2—O4^x^	106.57 (4)	O4^xiii^—Fe1—O1^iv^	92.17 (3)
O4^viii^—Ba2—O4^x^	73.43 (4)	O4^xiv^—Fe1—O1^iv^	92.17 (3)
O4^ix^—Ba2—O4^x^	180.0	O4—Fe1—O1^xv^	92.17 (3)
O4—Ba2—O3^ix^	111.55 (2)	O4^ix^—Fe1—O1^xv^	92.17 (3)
O4^viii^—Ba2—O3^ix^	68.45 (2)	O4^xiii^—Fe1—O1^xv^	87.83 (3)
O4^ix^—Ba2—O3^ix^	55.51 (2)	O4^xiv^—Fe1—O1^xv^	87.83 (3)
O4^x^—Ba2—O3^ix^	124.49 (2)	O1^iv^—Fe1—O1^xv^	180.0
O4—Ba2—O3^x^	68.45 (2)	O1—P1—O1^iii^	111.11 (9)
O4^viii^—Ba2—O3^x^	111.55 (2)	O1—P1—O2	109.59 (3)
O4^ix^—Ba2—O3^x^	124.49 (2)	O1^iii^—P1—O2	109.59 (3)
O4^x^—Ba2—O3^x^	55.51 (2)	O1—P1—O2^iii^	109.59 (3)
O3^ix^—Ba2—O3^x^	180.0	O1^iii^—P1—O2^iii^	109.59 (3)
O4—Ba2—O3^viii^	124.49 (2)	O2—P1—O2^iii^	107.28 (9)
O4^viii^—Ba2—O3^viii^	55.51 (2)	O3—P2—O3^xii^	112.84 (7)
O4^ix^—Ba2—O3^viii^	68.45 (2)	O3—P2—O4^xii^	112.49 (4)
O4^x^—Ba2—O3^viii^	111.55 (2)	O3^xii^—P2—O4^xii^	108.95 (5)
O3^ix^—Ba2—O3^viii^	77.32 (3)	O3—P2—O4	108.95 (5)
O3^x^—Ba2—O3^viii^	102.68 (3)	O3^xii^—P2—O4	112.49 (4)
O4—Ba2—O3	55.51 (2)	O4^xii^—P2—O4	100.50 (6)

Symmetry codes: (i) −*x*+1, −*y*+1/2, *z*−1; (ii) *x*, *y*, *z*−1; (iii) −*x*+1, −*y*+1/2, *z*; (iv) −*x*+3/2, −*y*+1, *z*−1/2; (v) *x*−1/2, *y*−1/2, *z*−1/2; (vi) −*x*+3/2, *y*−1/2, *z*−1/2; (vii) *x*−1/2, −*y*+1, *z*−1/2; (viii) −*x*+2, −*y*+1, −*z*+2; (ix) −*x*+2, *y*, *z*; (x) *x*, −*y*+1, −*z*+2; (xi) −*x*+3/2, −*y*+1/2, −*z*+3/2; (xii) −*x*+3/2, *y*, −*z*+3/2; (xiii) *x*, −*y*+1, −*z*+1; (xiv) −*x*+2, −*y*+1, −*z*+1; (xv) *x*+1/2, *y*, −*z*+3/2.
